# Establishing targets for advanced HIV disease: A call to action

**DOI:** 10.4102/sajhivmed.v22i1.1266

**Published:** 2021-08-10

**Authors:** David B. Meya, Lillian Tugume, Vennie Nabitaka, Proscovia Namuwenge, Sam Phiri, Rita Oladele, Bilkisu Jibrin, Mojisola Mobolaji-Bello, Cecilia Kanyama, Werner Maokola, Sayoki Mfinanga, Cordelia Katureebe, Ikechukwu Amamilo, Brian Ngwatu, Joseph N. Jarvis, Thomas S. Harrison, Amir Shroufi, Radha Rajasingham, David Boulware, Nelesh P. Govender, Angela Loyse

**Affiliations:** 1 Department of Research, Infectious Diseases Institute, Makerere University, Kampala, Uganda; 2 Department of Medicine and International Health, University of Minnesota, Minneapolis, United States of America; 3 HIV Department, Clinton Health Access Initiative, Kampala, Uganda; 4 Department of HIV Care and Treatment, Ministry of Health, Uganda, Kampala, Uganda; 5 HIV Department, Lighthouse Trust Malawi, Lilongwe, Malawi; 6 College of Medicine University of Lagos, Lagos, Nigeria; 7 Department of HIV Care, Treatment and Support, Ministry of Health, Lagos, Nigeria; 8 Department of Medicine, University of North Carolina Project-Malawi, Kamuzu Central Hospital, Lilongwe, Malawi; 9 National AIDS Control Program, Ministry of Health, Tanzania, Dar-es-Saalam, Tanzania; 10 Department of Research, Muhimbili Medical Research Centre, Dar-es-Salaam, Tanzania; 11 Department of National HIV Care and Treatment, Ministry of Health, Kampala, Uganda; 12 Global Health Access Program, Clinton Health Access Initiative, Abuja, Nigeria; 13 HIV Program, Clinton Health Access Initiative, Kampala, Uganda; 14 Department of HIV, London School of Hygiene and Tropical Medicine, London, United Kingdom; 15 Centre for Global Health, Institute for Infection and Immunity, St. George’s University of London, London, United Kingdom; 16 Department of HIV, Centres for Disease Control Foundation, Atlanta, United States of America; 17 Department of Research, National Institute for Communicable Diseases, Johannesburg, South Africa; 18 Department of Research, Centre for Global Health, Institute for Infection and Immunity, St. George’s University of London, London, United Kingdom

**Keywords:** advanced HIV disease, cryptococcal antigen, tuberculosis, TB-LAM, targets

## Abstract

The World Health Organization (WHO) has published a guideline for the management of individuals with advanced HIV disease (AHD) to reduce HIV-related deaths. The guideline consists of a package of recommendations including interventions to prevent, diagnose and treat common opportunistic infections, including tuberculosis (TB), cryptococcosis and severe bacterial infections, along with rapid initiation of antiretroviral treatment and enhanced adherence support. Currently no clear targets exist for these key interventions. Emerging programmatic data from Uganda, Tanzania and Nigeria suggest that an estimated 80% of eligible people continue to miss the recommended cryptococcal or TB testing, highlighting the remaining challenges to the effective implementation of WHO-recommended AHD packages of care in real-world resource-limited settings. The absence of mortality indicators for the leading causes of HIV-related deaths, because of the lack of mechanisms to ascertain cause of death, has had a negative impact on establishing interventions to reduce mortality. We suggest that setting 95-95-95 targets for CD4 testing, cryptococcal antigen and TB testing, and treatment that are aligned to the WHO AHD package of care would be a step in the right direction to achieving the greater goal of the WHO End TB strategy and the proposed new strategy to end cryptococcal meningitis deaths. However, these targets will only be achieved if there is healthcare worker training, expanded access to bedside point-of-care diagnostics for hospitalised patients and those in outpatient care who meet the criteria for AHD, and health systems strengthening to minimise delays in initiating the WHO-recommended therapies for TB and cryptococcal disease.

## Introduction

In 2017, the World Health Organization (WHO) published a guideline for the management of individuals with advanced HIV disease (AHD) (defined as having a CD4 count of < 200 cells/µL or HIV stage 3 or 4 disease in adults and adolescents or children younger than 5 years), as part of the strategy to reduce HIV-related deaths.^1^ This guideline outlines a package of care for persons with AHD and includes interventions to prevent, diagnose and treat common opportunistic infections (OIs), including tuberculosis (TB), cryptococcosis and severe bacterial infections, along with rapid initiation of antiretroviral treatment (ART) and enhanced adherence support.

Although the United States (US) Centers for Disease Control and the US President’s Emergency Plan for AIDS Relief facilitate implementation of the WHO guideline on the AHD package of care in many sub-Saharan African countries, especially in settings with a high number of persons with AHD, no clear targets exist for these key interventions to reduce OIs and mortality in persons with AHD. Such targets should include the proportion of persons accessing CD4 testing, persons with a CD4 count of < 200 cells/µL who are screened for TB and cryptococcal disease, and co-infected persons subsequently started on therapy (Table 1). These targets are akin to the broader 95-95-95 targets of the Joint United Nations Programme on HIV/AIDS (UNAIDS): to achieve by 2030 95% of persons tested for HIV, 95% of those HIV-positive started on ART and 95% of those on ART attaining virological suppression.

**TABLE 1:** The basic indicators for advanced HIV disease care.

Indicator	Reason for monitoring and evaluation
Number of persons with a new HIV diagnosis; number of persons with an HIV diagnosis returning to care; number of persons on ART without HIV viral suppression	These categories of persons are at high risk of subclinical OIs and require a CD4 test (< 200 cells/mL) and/or clinical evaluation to determine whether they may have a Stage 3 or 4 illness – to identify them as having advanced HIV disease.
Number of persons in the above categories receiving cryptococcal antigen testing	To determine persons who require treatment and further evaluation for cryptococcal meningitis.
Number of persons in the above categories receiving urine TB-LAM testing	To determine persons who require treatment and further evaluation for disseminated TB.
Number of persons with evidence of cryptococcal or TB infection(s) who receive appropriate treatment	To determine linkage to treatment for TB and cryptococcal disease.

ART, antiretroviral treatment; TB-LAM, tuberculosis lipoarabinomannan; OIs, opportunistic infections.

Despite the reduction in HIV-related deaths by 39% since 2010, nearly 700 000 HIV-related deaths occurred in 2019,^2^ with HIV-related illness remaining the leading cause of death in sub-Saharan Africa. Notwithstanding the rapid roll-out of ART globally, data from South Africa show that about a third of individuals entering or cycling in and out of HIV care have advanced disease, with minimal change over the last decade.^3^ Similarly, in Uganda and Botswana approximately 20% – 25% of persons entered care with advanced HIV in 2020.^4,5^ Considering that most AHD is diagnosed among ART-experienced persons,^6^ ART non-adherence with treatment failure will remain a significant contributor to incident OIs despite expanded ART initiation.^7^

Based on the WHO guidance, each person diagnosed with HIV should have a CD4 test performed.^1^ Of those with a CD4 count of < 200 cells/µL, cryptococcal antigen (CrAg) and TB testing should be offered using a CrAg lateral flow assay (LFA) and Xpert/TB lipoarabinomannan (LAM) assay.^1^ Those with evidence of disease (i.e. positive tests) should be started on appropriate treatment; for example, TB preventive therapy should be started for those with latent TB infection. In many countries, some steps in this cascade are not always executed. Multiple reasons can exist for this failure to implement, including basic stock-outs of CD4 reagents, diagnostic test kits and the medicines required for OI treatment.^8^ There is a significant deleterious impact on AHD outcomes when only some of these resources and not all are available. In some cases, the implementation of these interventions is not prioritised by healthcare workers, who may have limited training on AHD management^9^ and consider these screening tests as less imperative compared to initiating ART. In addition, weak health systems may result in greater attrition of persons entering HIV care even after a CD4 test, in the absence of tracking mechanisms to complete this cascade of care when they fail to return.

Many national programmes lack mortality indicators for the leading causes of HIV-related deaths, which is partly a result of poor data around ascertaining a cause of death. Data from the last quarter of 2020 from the AIDS Control Programme of the Ministry of Health in Uganda^10^ showed that only 36% of newly diagnosed HIV-infected patients received a CD4 test. Of these, 25% met the definition of AHD with a CD4 count of < 200 cells/µL. Subsequently, of those with AHD, 61% received CrAg testing (8% CrAg test positive), and 89% of CrAg-positive persons were then started on pre-emptive fluconazole. For TB screening, only 56% of those with a CD4 count of < 200 cells/µL received urine LAM testing (19% positive for urine LAM), and 92% of these received TB treatment. Overall, this extrapolates to an estimated 80% having missed the recommended cryptococcal or TB screening. Similarly, among persons with virological failure, 6% received CD4 testing, with 25% having a CD4 count of < 200 cells/µL. Among those with CD4 testing, 73% received CrAg testing (12% CrAg test positive; 82% treated), and 74% received urine TB-LAM testing (20% urine LAM test positive; 80% treated).^11^ A gap in knowledge remains for the OI prevalence among those persons returning to care after attrition. There are challenges in obtaining data on longer-term disease outcomes among persons who screened for these OIs during the cascade of AHD management. However, cryptococcal survival outcomes remain 30% – 40% better when CrAg-positive persons are pre-emptively treated while asymptomatic, rather than treating symptomatic meningitis.^12^

Data from a retrospective cohort in Mwanza, Tanzania, of 700 participants showed that 261 (35%) had WHO stage 3 and 4 HIV disease, and 258 (35%) had baseline CD4 counts of < 200 cells/µL.^13^ These programmatic data in Tanzania show a similar trend to data reported from Uganda. Among persons who initiated ART in 2018, 21% had WHO clinical stage 3 or 4, and 31% had a CD4 count of < 200 cells/µL.^14^ In this cohort, the mortality rates decreased with increasing CD4 count at enrolment. Persons with a CD4 count of < 200 cells/µL had a high mortality rate of 61.69 per 1000 person/year, compared to the mortality rate of 18.01 per 1000 person/year for those enrolled with CD4 counts of ≥ 350 cells/µL (mortality rate ratio: 3.43). The data also showed a higher mortality rate of 117.03 per 1000 person/year for those enrolled with WHO clinical stage 4, compared to the mortality rate of 53.25 per 1000 person/year for patients enrolled with WHO clinical stage 3 (mortality rate ratio 2.20). The Nigerian national 2020 data for people living with HIV and on active TB on treatment is 83.5%,^15^ while there are currently no data from the Nigeria repository on CrAg and urine TB-LAM testing. However, preliminary data from a pilot AHD implementation project at a large ART centre (Lagos University Teaching Hospital, Lagos, Nigeria) revealed that from August 2020 to January 2021, 47.9% (69/144) of newly enrolled people living with HIV had a CD4 count of < 200 cells/µL, with all receiving reflex CrAg screening and 65 (94.2%) receiving TB lateral flow LAM testing.

Sub-Saharan Africa bears the brunt of HIV-associated OIs, with an estimated 162 500 cases of cryptococcal meningitis in 2014 (73% of the global burden) and 135 900 deaths (75% of the estimated global burden).^16^ In 2019, of the 1.4 million TB-related deaths that occurred, 208 000 were among people living with HIV.^17^ The WHO End TB strategy is designed with the aim of achieving a 90% decrease in TB deaths by 2030.^18^ As a step towards achieving this goal, it is critical that persons with AHD be identified, screened and treated for TB in a timely manner. Therefore, we suggest that setting targets for CD4 testing, CrAg and TB testing, and treatment, that are aligned to the WHO AHD package of care would be a step in the right direction to achieving the greater goal of the WHO End TB strategy and the proposed new strategy to end cryptococcal meningitis deaths.^19^

Efforts should be aimed at ensuring that all individuals recently diagnosed with HIV, those returning to care or those who are not virologically suppressed have access to CD4 testing. Prompt identification of persons at high risk in the setting of AHD remains important for minimising poor outcomes. The roll-out of the WHO-approved point-of-care VISITECT CD4 Advanced HIV Disease LFA by Omega Diagnostics (Alva, United Kingdom) provides an instrument-free, semi-quantitative result for CD4 (greater than or less than 200 cells/µL), which should be rapidly expanded; however, feasibility studies for point-of-care use in routine care settings are needed to inform placement and scale-up. Unitaid and the Clinton Health Access Initiative have launched an early market access vehicle to provide access to this test at no cost in over 130 countries to determine operational feasibility in routine care settings.^20,21^

**FIGURE 1:**
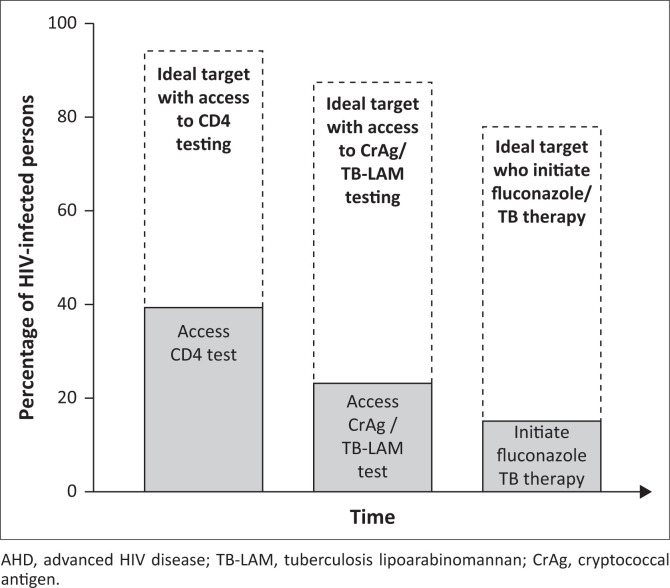
Schematic detailing comparison between ideal AHD targets and current AHD indicator performance.

We further recommend that national HIV programmes set 95-95-95 targets for 95% of persons with a new HIV diagnosis, those returning to care or non-suppressed populations to receive CD4 testing, 95% of those with a CD4 count of < 200 cells/µL to be screened with CrAg and TB LAM, and 95% testing CrAg or LAM positive to be treated for those by 2030 (Figure 1). These targets would apply to both outpatient and inpatient settings and spur the design and implementation of real-world interventions to reduce the morbidity and mortality complicating AHD. In addition, these targets will drive countries to establish mechanisms for tracking these targets as well as AHD-related mortality within their national reporting systems. We believe that these targets are achievable, given the progress towards the even more challenging UNAIDS 95-95-95 targets set for 2030.

In a CrAg screening model for Uganda, Rajasingham and colleagues suggested that CrAg screening and treatment (assuming a national CrAg prevalence of 1.4%) would save 7320 lives at a cost of $459.00 per life saved.^22^ In contrast, the cost of treating a person with AHD who develops cryptococcal meningitis using the current WHO-recommended regimen of amphotericin B and flucytosine for 1 week is $1861.00,^23^ making CrAg screening and fluconazole pre-emptive therapy a more cost-effective approach. Menzies and colleagues similarly showed that expanding TB diagnostics and care access produced substantial health gains to achieve the goals set out in the End TB strategy, based on an analysis of nine cost-effectiveness models in China, India and South Africa.^24^ We posit that these gains and cost savings can only be maximised if the AHD package is fully implemented with everyone who should receive CD4, CrAg and TB-LAM testing, with the appropriate therapy for those diagnosed with disease. For instance, cost savings for CrAg screening would be maximised by screening individuals with a CD4 count of < 200 cells/µL with an expected prevalence of 6% – 7%^16^ as opposed to 1.4% in the general HIV population. This can be enhanced if point-of-care CD4 testing is implemented adequately as the next step following HIV testing. In order to achieve this, it is imperative that procurement and supply systems for the resources required, are coordinated efficiently while anchored within strong health systems and regular healthcare worker training. Furthermore, implementation research is still required to improve clinical outcomes among patients with AHD, for example by evaluating the effectiveness and feasibility of performing lumbar punctures in those with a positive CrAg test result.

The $20 million investment by Unitaid through 2022 to avert preventable deaths among persons with AHD in eight African low- and middle-income countries and India is certainly a step in the right direction.^25^ Participating countries should utilise the catalytic procurement of commodities to generate local evidence and leverage the programmatic support during the project period to ensure that AHD interventions are sustainable. Furthermore, these interventions need to be expanded to other countries in sub-Saharan Africa and beyond 2022 through increased and coordinated demand for commodities through entities like institutional donors, along with expanded regular training and health systems strengthening.

Expanded access to bedside point-of-care diagnostics for hospitalised patients to minimise delays in initiating standard-of-care therapies (for TB and cryptococcal disease) would improve survival. However, waiting for persons with AHD to be hospitalised exposes weaknesses in the health system when ambulatory HIV care neglects essential interventions to prevent AHD complications. By prioritising training, mentorship and health systems strengthening such that outpatient clinicians have excellent clinical skills and are empowered with point-of-care diagnostics (e.g. CD4, CrAg, TB-LAM) and adequate resources for optimal treatment, the proposed targets could be met, resulting in potentially fewer hospitalisations and AHD-related mortality. While OIs will continue to occur screening and pre-emptively treating OIs at an early stage in an outpatient setting is far less expensive than hospital care and results in better outcomes.

We believe that establishing and implementing country-specific interventions to ensure that 95% of persons with a new HIV diagnosis or those without HIV viral suppression receive CD4 testing, 95% of persons with a CD4 count of < 200 cells/µL receive CrAg and TB-LAM testing, and 95% of those with positive CrAg and LAM tests initiate prompt treatment would go a long way in reducing HIV-related mortality. Creating targets and ensuring accurate documentation of the metrics including AHD-related mortality would help national programmes focus efforts on addressing interventions to meet targets for optimal AHD care to minimise HIV-related deaths.
